# High-throughput identification of viral termini and packaging mechanisms in virome datasets using PhageTermVirome

**DOI:** 10.1038/s41598-021-97867-3

**Published:** 2021-09-15

**Authors:** Julian R. Garneau, Véronique Legrand, Martial Marbouty, Maximilian O. Press, Dean R. Vik, Louis-Charles Fortier, Matthew B. Sullivan, David Bikard, Marc Monot

**Affiliations:** 1 Biomics Platform, C2RT, Institut Pasteur, 75015 Paris, France; 2 Infrastructure et Ingénierie Scientifique, Institut Pasteur, 75015 Paris, France; 3 Institut Pasteur, Unité Régulation Spatiale des Génomes, UMR 3525, CNRS, 75015 Paris, France; 4 Phase Genomics Inc, Seattle, WA 98109 USA; 5 Department of Microbiology, Ohio State University, Columbus, OH 43210 USA; 6 Faculty of Medicine and Health Sciences, Department of Microbiology and Infectious Diseases, Université de Sherbrooke, Sherbrooke, QC J1E 4K8 Canada; 7 Département de Microbiologie, Institut Pasteur, Groupe Biologie de Synthèse, 75015 Paris, France

**Keywords:** Genomics, Sequencing, Computational biology and bioinformatics, Data processing, Genome informatics, High-throughput screening, Software, Microbiology, Bacteriophages, Phage biology, Virology

## Abstract

Viruses that infect bacteria (phages) are increasingly recognized for their importance in diverse ecosystems but identifying and annotating them in large-scale sequence datasets is still challenging. Although efficient scalable virus identification tools are emerging, defining the exact ends (termini) of phage genomes is still particularly difficult. The proper identification of termini is crucial, as it helps in characterizing the packaging mechanism of bacteriophages and provides information on various aspects of phage biology. Here, we introduce PhageTermVirome (PTV) as a tool for the easy and rapid high-throughput determination of phage termini and packaging mechanisms using modern large-scale metagenomics datasets. We successfully tested the PTV algorithm on a mock virome dataset and then used it on two real virome datasets to achieve the rapid identification of more than 100 phage termini and packaging mechanisms, with just a few hours of computing time. Because PTV allows the identification of free fully formed viral particles (by recognition of termini present only in encapsidated DNA), it can also complement other virus identification softwares to predict the true viral origin of contigs in viral metagenomics datasets. PTV is a novel and unique tool for high-throughput characterization of phage genomes, including phage termini identification and characterization of genome packaging mechanisms. This software should help researchers better visualize, map and study the virosphere. PTV is freely available for downloading and installation at https://gitlab.pasteur.fr/vlegrand/ptv.

## Introduction

Viruses play key roles in diverse microbial ecosystems. They are recognized to influence biogeochemical cycling, modulate microbial populations and metabolism, and act as a driving force in gene flow in soil and the oceans^1–6^. In humans, viruses can also provide a first line of non-host derived immunity^7^ and are associated with health status^8–12^. Such discoveries have led to an increasing interest in characterizing viruses and their impact on ecosystems. However, although metagenomic surveys have revealed hundreds of thousands of viral genomes and large genome fragments^13–18^, these new genomes likely only scratch the surface of the viruses existing in these environments^15,18^.

A considerable proportion of the reads obtained when sequencing the human gut virome typically has no significant homology to known viral genomes^19^. For example, Manrique et al*.* reported that only a small subset of active phages detected in the gut microbiome could be taxonomically classified and that more than half (≈ 60%) could represent entirely unknown novel bacteriophages. Similarly, datasets from the deeply sequenced Global Ocean Viromes show that only a minimal fraction of reads (≈ 10–20%) can be mapped to large de novo assembled viral reference genome databases, such as GOV1 and GOV2 databases^13,14^. The term “viral dark matter” has been used to describe this vast unknown sequence space^19–21^. Recent efforts to establish viral clusters in gene-sharing networks have revealed structures that will greatly help in virus identification and classification, but a large number of unknown clusters likely remain to be discovered^22,23^. Despite the continuous rise in the number of sequences compiled in these databases, researchers will undoubtedly keep finding unknown sequences in abundance for the foreseeable future. It is thus still important to ascertain the viral origin of sequences discovered in metagenome analysis with higher confidence. Phage and virus biologists have therefore focused their efforts on developing orthogonal complementary prediction approaches, such as the analysis of k-mer frequencies and viral genome features, also recently implemented through machine-learning algorithms^24,25^.

It has been previously shown that the ends of linear DNA, a hallmark of genomic DNA (gDNA) packaged in the capsid of free phage particles, can be easily detected from shotgun sequencing data if the sequencing library was prepared in a way that preserves such DNA ends (i.e., random fragmentation of DNA prior to library preparation)^26,27^. Shotgun sequencing approaches typically rely on the ligation of adapters to randomly fragmented DNA. During this process, DNA fragments with one end that corresponds to natural genome termini will be strongly enriched relative to other DNA fragments that end at random positions due to the random fragmentation of DNA during library preparation. This enables the recognition of termini by analyzing the number of reads that start at each position along a given phage sequence, normalized against the whole sequence coverage. The number of read starts mapping precisely at the natural termini will be over-represented, because it directly correlates with the number of genomes (i.e., viral particles) present in a sample. We previously used this concept and approach to identify the genome termini and characterize packaging mechanisms of individual pure phages^27^.

Here, we introduce PhageTermVirome (PTV), an easy-to-use bioinformatics software that efficiently identifies critical information such as phage genome termini and packaging mechanisms in modern large-scale viral metagenomics datasets. Due to its ability to detect termini present in capsid-packaged linear DNA, PTV also offers an entirely orthogonal and complementary approach to predict the true viral origin of metagenomic contigs, along with other existing phage prediction tools^28–36^.

## Results and discussion

### Overview of the PhageTermVirome workflow

**Figure 1:**
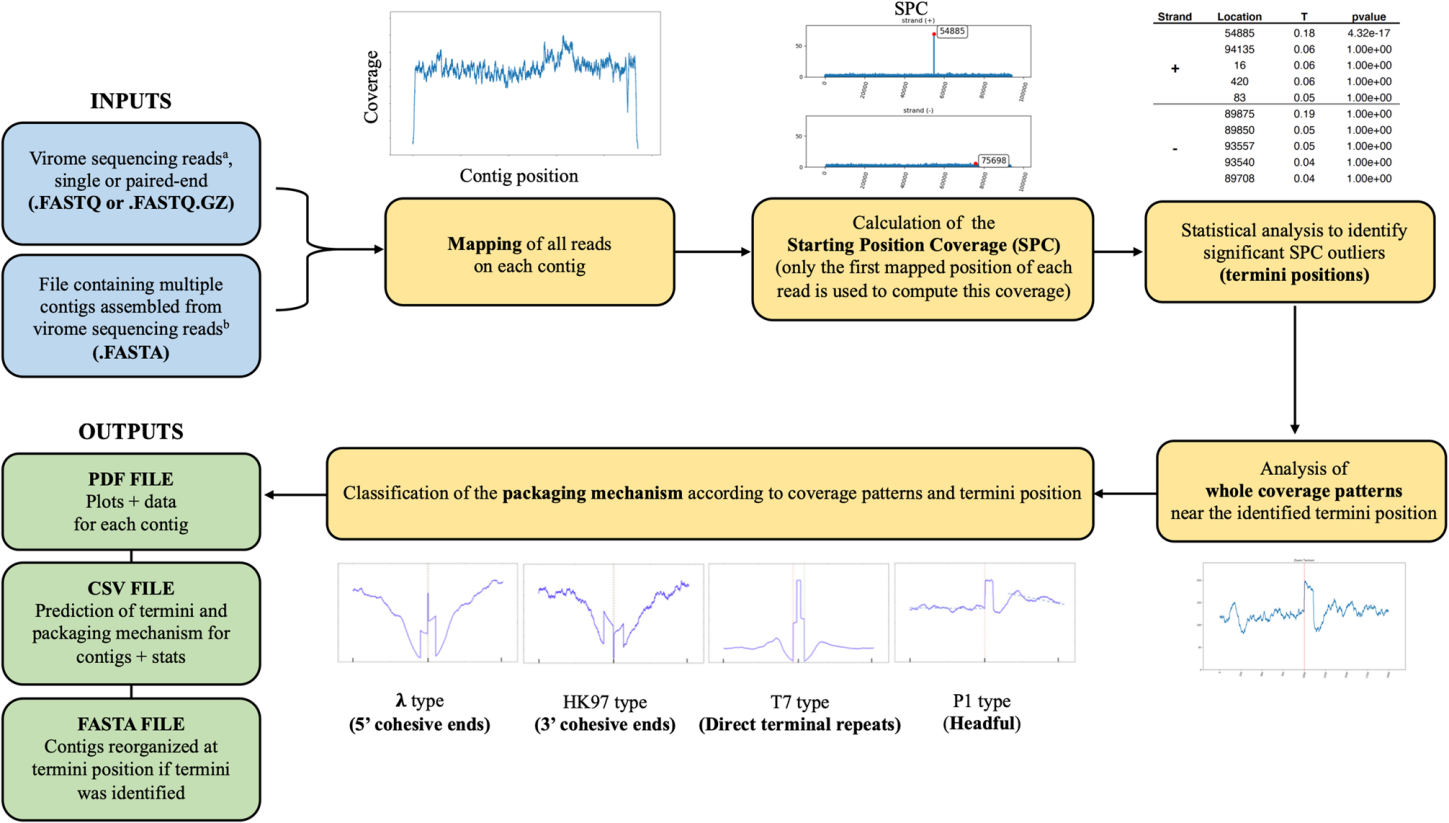
Overview of the different steps involved in the PhageTermVirome workflow. Virome sequencing reads are simultaneously mapped to numerous contigs assembled from the virome dataset. After alignments for each contig, PTV performs peak detection and statistical analyses to determine the position of significant termini. On all contigs for which termini were detected, a coverage pattern analysis is achieved to predict the corresponding genome packaging mechanism. ^a^The sequencing method and library preparation require the preservation of genome ends (ex: all methods involving random DNA fragmentation prior to library preparation and long-read sequencing of intact DNA). ^b^PhageTermVirome can process massive amount of contigs as input (multifasta), but still allow users to analyze a single phage contig (fasta).

The key steps of the PTV analysis workflow are depicted in Fig. 1. They consist of: (i) mapping all viral metagenomic reads on contigs assembled from the same read dataset, (ii) calculation of the starting position coverage (SPC) and identification of positions on the contig for which the SPC is significantly higher, which may represent genome ends (termini) and secondary terminase cutting sites, (iii) if applicable, characterization of the corresponding packaging mechanism by analysis of the coverage patterns near the identified termini, and (iv) aggregation of the prediction results and coverage plots for all contigs submitted to PTV into a single PDF file for visual inspection. A CSV file containing the predictions and statistics for each contig is also provided to users for easier consultation and manipulation of the results in a table format, which can be useful when there is a very large number of contigs. For contigs with successful predictions, PTV also outputs a new fasta file containing the contigs reorganized to start at the predicted termini.

The PTV algorithm was developed to allow the analysis of multiple contigs in a single workflow (i.e., users do not have to re-run the script for each contig they want to analyze). The mandatory inputs into PTV required for analysis are (i) a multifasta (.fasta) file containing the assembled contigs and (ii) a fastq file (.fastq) containing the reads used to assemble the contigs found in the fasta file. When available, the user can also provide the corresponding paired-end fastq read file. Although not required, the use of paired-end reads is recommended, as it has been shown to improve termini and packaging mechanism prediction^27^.

The PTV algorithm is largely based on the mapping of all reads of a dataset to each contig provided, which can represent a daunting task for ultra-deeply sequenced viromes containing a vast amount of reads and a large number of contigs. Additional options and parameters have thus been added to the PTV scripts to accelerate and scale the process. For example, in addition to the multi-threading option initially implemented in the original PhageTerm software^27^ (using the command-line option --core), a new multi-machine functionality has also been deployed in PTV (using the command-line option --mm). The multi-machine option can be used to go over the limit of the number of cores available on a machine when processing large datasets and thus accelerate the analysis. It is intended for advanced users who have the possibility to perform analyses on multi-machine computer clusters. As PTV can still rapidly process average-sized virome datasets using only multi-threading options, the multi-machine option becomes more attractive for extra-large datasets (e.g., ≳ 100 million reads with ≳ 1000 contigs). Many factors can influence the required time to complete a PTV analysis, such as the total number of reads, the total number and length of contigs to analyze, the speed of the processors, and the number of cores available for parallelization.

### Detection of phage termini and packaging mode in a control mock virome dataset

**Figure 2:**
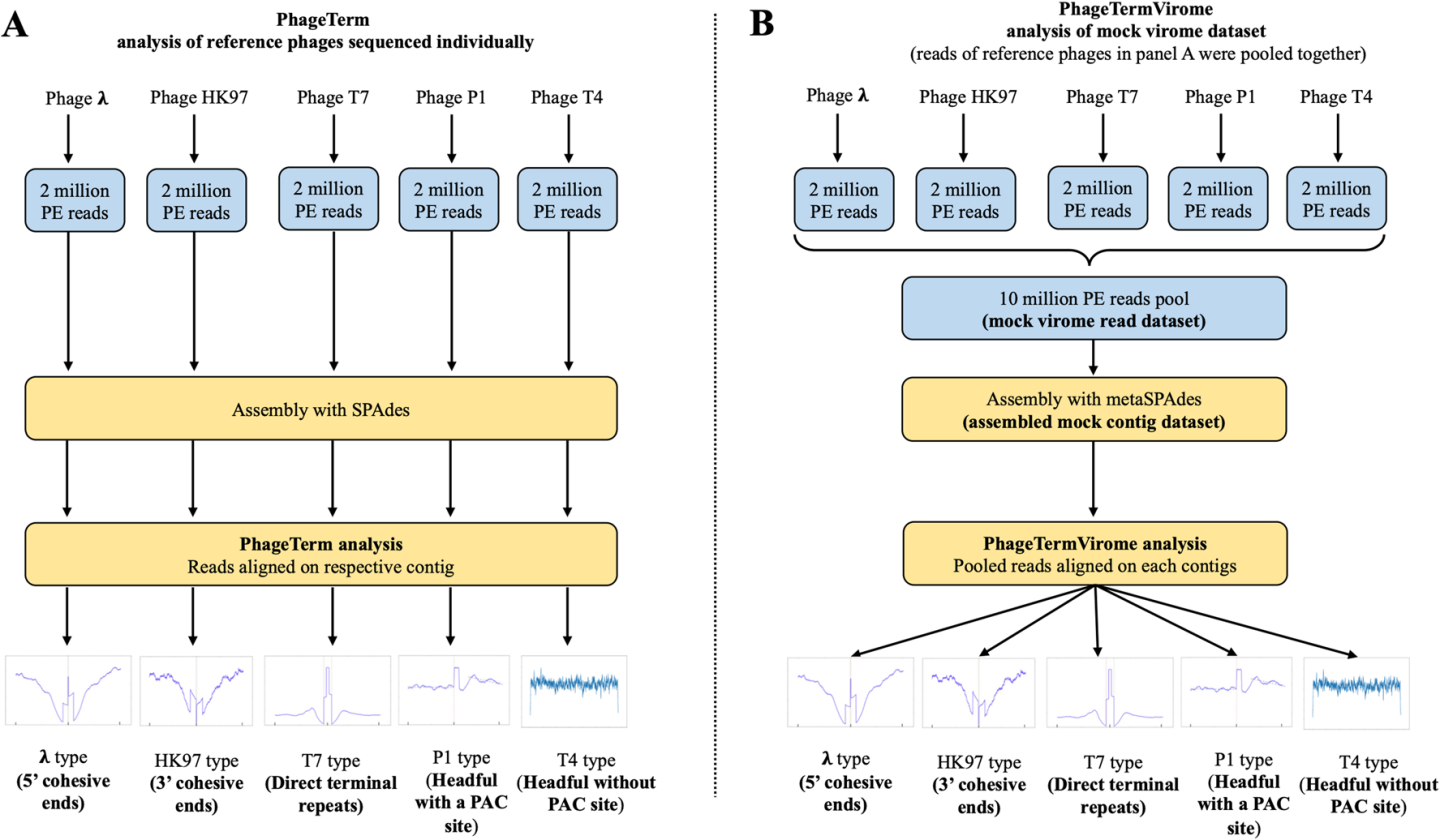
Comparison of results obtained with PhageTerm versus PhageTermVirome when using a mock virome dataset. (**A**) Individually sequenced phages, separately analyzed using PhageTerm previously confirmed the well-established packaging mechanism for each reference phage. (**B**) Reads of reference phages used in panel (**A**) were pooled to form the mock virome read dataset and were then assembled with metaSPAdes to generate the associated mock viral contig dataset. The pool of 10 million reads were mapped to the assembled contigs to obtain coverage values, statistics, termini positions, and packaging mechanism predictions for each contig.

We first assessed the ability of PTV to correctly identify termini and packaging modes in metagenomics datasets using a mock viral metagenomics dataset. This dataset was built by pooling 1 × 10^7^ paired-end reads from five reference phages, using 2 × 10^6^ paired-end reads from each phage previously sequenced individually^27^. HK97, T4, T7, P1, and Lambda phage paired-end reads were used, as these phages harbor a diversity of well-described termini and packaging mechanisms. After running the PTV workflow on the mock read dataset and the reference phage genomes, the expected termini positions were identified and corresponding packaging mechanisms successfully defined by PTV for all five reference phages (Fig. 2): Lambda (5′cos), HK97 (3′cos), P1 (headful with pac site), and T7 (direct terminal repeat, DTR). As expected, PTV returned no signal for T4 (headful without precise pac site). It is known that T4 type phages generate no precise or detectable termini, as their DNA is randomly packaged inside the capsid. This type of phage packages full genomes with redundant ends, starting and ending at random positions, generating no observable over-represented sequencing biases after sequencing. Our results on the mock virome dataset suggest that PTV algorithms, coverage analyses, and statistical models are sufficiently robust (assuming sufficient coverage) to accurately detect termini and classify packaging mechanisms when the reads of different phages are merged, as is the case for real virome metagenomic datasets.

### Characterization of phage termini and packaging mode in two real virome metagenomics datasets

**Figure 3:**
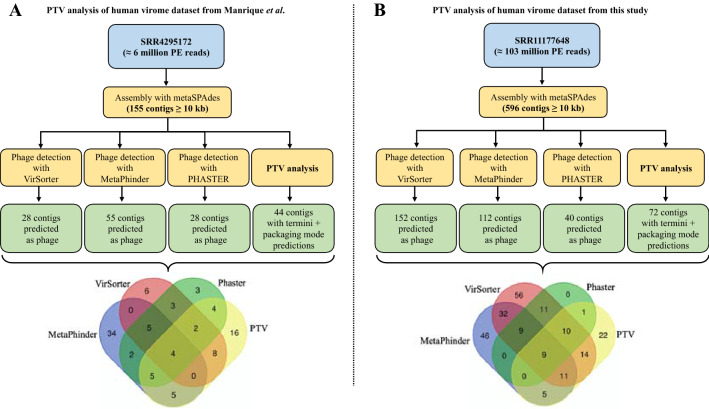
PhageTermVirome analysis with two human virome datasets. (**A**) PTV analysis of 155 contigs assembled from the study of Manrique et al. (**B**) PTV analysis of 596 contigs assembled from the virome dataset generated for this study.

Given the successful characterization of phage termini and packaging mechanisms in the mock virome dataset, we next applied PTV to two real virome datasets. The first dataset selected to test PTV was a human gut virome previously sequenced and published in the study by Manrique et al.^37^. The second dataset was generated during the course of this study and also corresponds to a human gut virome (Fig. 3). In addition to analyzing the two virome datasets with PTV, we also performed a phage prediction analysis with three widely used software programs, MetaPhinder^30^, VirSorter^29^ and Phaster^31^. Concomitant analysis of the assembled contigs was performed with these software programs and PTV to verify whether our tool performs better on contigs that are typically predicted to be phages. Thus, we produced Venn diagrams showing contigs individually and commonly predicted to be phage by each tool and compared the results with the successful termini PTV predictions.

For the Manrique dataset, PTV predicted termini and the packaging mode for 44 contigs from a total of 155 assembled contigs. Among the 44 contigs, 28 were predicted to be phage by the other softwares, meaning that PTV could determine termini and the packaging mode on contigs (N = 16) that were not declared to be phage by any other prediction tools. Detailed analysis of these 16 contigs showed a diversity of predicted packaging modes (4 *cos* phages, 10 *pac* phages, and 2 DTR phages).

In the second human virome dataset, generated for this study, PTV was able to predict termini and the packaging mode for 72 contigs (from a total of 596), among which 50 were also predicted to be phage by the other phage prediction tools. Thus, PTV obtained predictions for a number of contigs (N = 22) in this dataset that were not identified as phage by any of the other tools. The predicted packaging modes for these 22 contigs were also diverse, with 8 *cos* phages, 8 *pac* phages, and 6 DTR phages. One finding that arises from this comparative analysis is that each phage prediction software performs unevenly on different datasets and that it is useful, even necessary, to use a combination of different tools to obtain a more complete and precise vision of the landscape of the phages in a virome dataset.

In light of these results, we wished to understand why certain contigs with successful PTV predictions could not be established as phage by any of the three prediction tools used in this study. Thus, the 38 contigs (16 + 22) were annotated with PROKKA using the latest PHASTER phage protein database (as of December 2020). For each contig, if an ORF was not annotated with a known protein in the database, a second annotation was performed using PROKKA’s default bacterial protein database. Proteins of phage origin could be found on most of the contigs but, in many cases, represented a minority of the total predicted ORFs. The high number of hypothetical proteins found on these contigs likely explains, at least partially, why they were not identified as phage by the other prediction tools used here. Another hypothesis is that these contigs may be fragments of a partially assembled phage genome, as is often the case in virome metagenomic datasets^38^. In such fragmented assemblies, hallmark phage genes required by certain tools to declare the contig as phage may be lacking. However, given sufficient read coverage, PTV is still able to determine termini and packaging modes on a contig, even if a large part of the corresponding phage genome is missing from the assembly. This feature also allows PTV to discriminate between sequences of active phages and those of purely decaying or inactive prophages (which can be present in contaminating bacterial genomes, but which ultimately do not form viral particles packaging linear DNA with termini). It is difficult to confirm the completeness of a phage contig obtained after the sequencing of a virome sample, especially when using short-read sequencing. To date, the most reliable way to ensure full genome recovery is still probably the sequencing of pure cultured phage lysates. Sequencing of virome DNA using long-read technologies also shows great promise in retrieving more complete phage genomes^39^.

**Figure 4:**
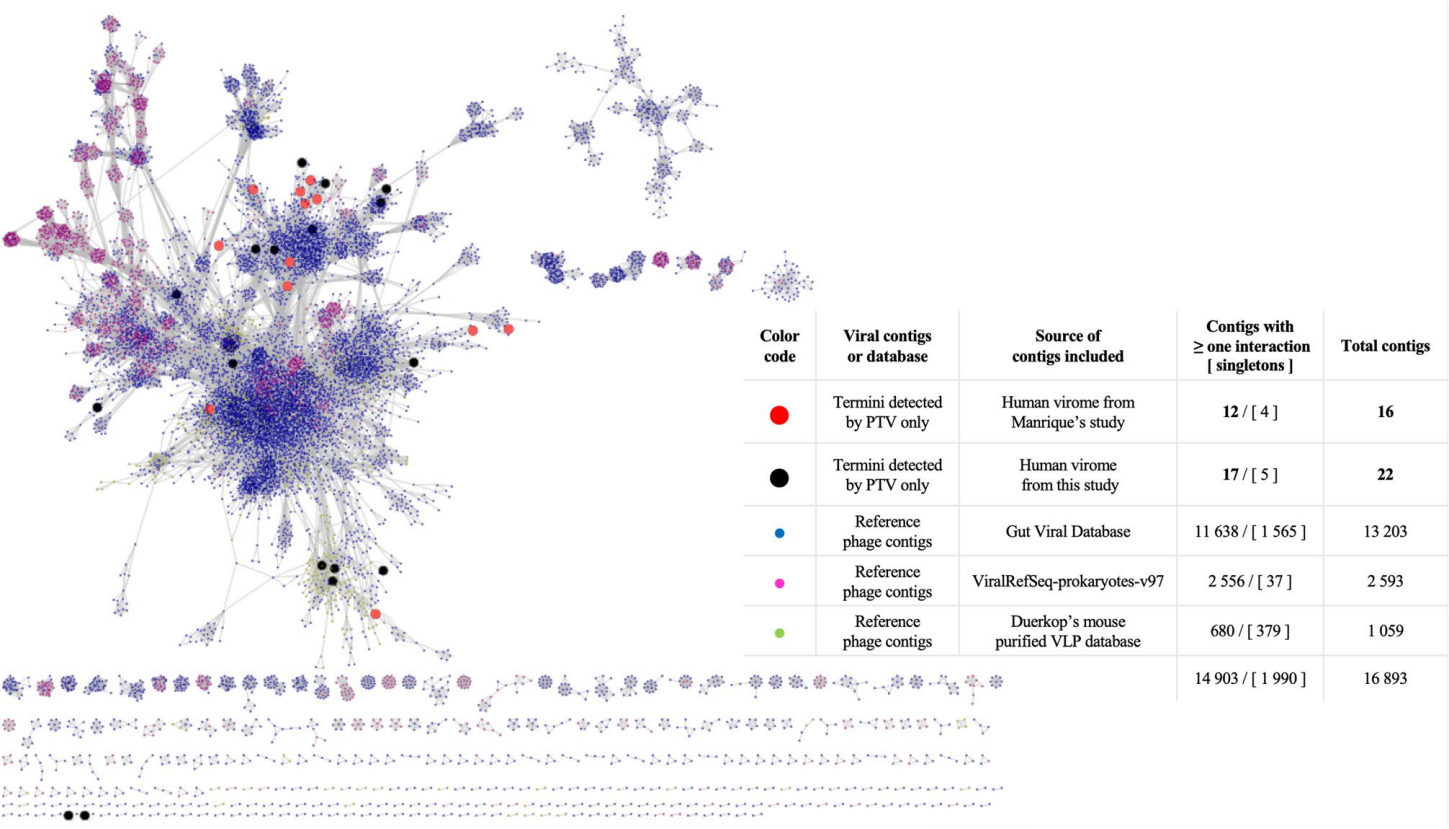
Viral cluster analysis with vConTACT2 using a gene-sharing network. The large red dots represent phage contigs (Manrique dataset) for which PTV predicted a terminus and packaging mode but not predicted to be viral by MetaPhinder, Phaster, or VirSorter. The large black dots represent phage contigs (dataset from this study) for which PTV predicted a terminus and packaging mode but not predicted to be viral by MetaPhinder, Phaster, or VirSorter. Network analysis was performed using three reference contig collections: GVD^8^, ViralRefSeq V.97^41^, and a database of curated virus-like particles from mice^42^.

Since only a few phage proteins could be identified after annotation of our contigs for which we obtained a termini and packaging mode prediction, we sought to perform a more in-depth analysis using vConTACT2^23^ in order to obtain additional clues about the taxonomy of those contigs (Fig. 4). In this analysis, 12 of 16 contigs from the Manrique dataset clustered with at least one contig from the reference viral databases included here. It also showed that 17 of 22 contigs from the dataset generated for the study also clustered with contigs from the reference viral databases. Further in-depth analyses of the contigs found to be singletons will help determine the nature of these sequences and whether they represent unknown and distant phages or other entities capable of packaging DNA, such as phage-inducible chromosomal islands (PICIs), genomic islands, or other classes of transducing agents^40^.

### Limitations and perspectives for PhageTermVirome

The capacity of PTV to detect termini and identify packaging mechanisms depends on the protocol used to prepare the nucleic-acid libraries prior to sequencing. Indeed, the detection of DNA termini can only occur if they are preserved during library preparation. Methods such as tagmentation, used for example in the Nextera kits from Illumina, insert adapters within DNA fragments in a non-random fashion (creating non-natural sequencing biases for read start positions) and are therefore incompatible with the natural bias-detection procedure of PTV. As previously mentioned, to enable data analysis using PTV, the sequencing library must be prepared through the ligation of adapters to DNA that has been randomly fragmented (e.g., Covaris, sonication), or any DNA preparation in which the natural DNA ends have been preserved. (e.g., Illumina TruSeq PCR free and TruSeq Nano, NebNext, Accel-NGS 1S PluS DNA library Kit). Note that the approach is constrained by the sequencing library preparation methods but not the sequencing technology itself. Although only Illumina sequencing was assessed here, other sequencing technologies or approaches, such as SMRT PacBio sequencing, Nanopore sequencing, and the recently developed VirION2 approach^43^, are theoretically also well suited to work with PTV. These sequencing technologies exhibit higher error rates than short-read sequencing approaches, but this should not strongly affect the ability of PTV to detect termini and determine packaging mechanisms. The first step of the PTV pipeline is based on the perfect alignment of only short seed sequences, of which the length can be adjusted if needed (default is 20-mers at the start of each read).

It is important to keep in mind that the algorithm of PTV is based on read alignments and the detection of over-represented sequences. Thus, the performance of PTV may be affected by very low coverage depth. Users must keep in mind that PTV will make more confident calls for contigs with a reasonable mean coverage. PTV can work at low mean coverage (e.g., 10 ×) in some cases, but often requires higher mean coverage for the unequivocal detection of termini and packaging mechanism (e.g., around 30 ×). Coverage depth can be variable from one contig to another in virome datasets and can be very low for viral particles that are scarce in the sample. PTV can often identify the position of potential termini for contigs with very low coverage, but may have difficulty in properly determining the packaging mechanism. PTV issues a warning for contigs exhibiting very low coverage and invites users to manually inspect the results and consult the coverage-pattern plots to confirm ambiguous results.

For extra-large virome datasets (a massive number of reads and a very large quantity of contigs), PTV analysis may require long processing times, especially if the analysis is being performed with very few computing cores. In this first version of PTV, a multi-threading option is available, as well as a newly implemented multi-machine option. When parallelization is not possible, alternatives can be used to reduce preventable computing time, such as excluding undesired or irrelevant contigs (e.g., very short contigs ≤ 1 kb or other contigs representing non-viral contamination). The minimal contig limit size can be customized in PTV using the “--limit” option (default is 500 bp). However, as previously mentioned, we expect PTV to be useful for termini detection and characterization for entities known to package small non-random DNA fragments (e.g., *Dinoroseobacter shibae* gene transfer agents can package 4.2 kb dsDNA^44^. Other examples include particles packaging very small linear DNA or RNA fragments, such as satellite DNA or RNA viruses, as well as linear satellite RNA (satRNA). Group 1 large single-stranded satRNAs can package linear RNA of 0.7–1.5 kb and group 2 single-stranded satRNAs typically package linear RNA fragments < 700 bp (21,994,595). Thus, we recommend carefully choosing the metagenomics contigs excluded from the analysis. Another recommendation, also left to the discretion of the user, is to pre-select contigs with a minimal coverage threshold (e.g., reject contigs ≤ 10 × mean coverage), which will reduce the processing time and also help to generate more robust results.

The PhageTerm approach is naturally restricted to the detection of viruses that package linear nucleic acids. In viral metagenomic samples, dsDNA phages of the caudovirales order represent most sequences known to date, but a large number of diverse phages are yet to be discovered. Recent studies have shed light on the previously underestimated prevalence of single-stranded DNA (ssDNA) phages, including *Innoviridae* and *Microviridae* phages^45–47^, but the ssDNA viruses discovered thus far appear to package circular DNA and therefore escape detection and characterization by PTV. As certain eukaryotic viruses can package linear ssDNA with inverted terminal repeats, such as Parvoviruses and Bidensoviruses^48,49^, we have to consider the possibility that linear ssDNA phages may also be found in nature and that they may be detected and characterizable by PTV. Our strategy is also not restricted to bacteriophages. Thus, viruses that package linear nucleic acids that infect any kingdom, including ssDNA viruses and linear RNA viruses, should also be characterizable by PTV. The detection of the termini of linear RNA viruses requires custom sample preparation protocols which were not explored here. Protocols akin to those used to identify transcription starts in mRNA sequencing could readily be used to identify the 5′ end of RNA viruses^50^, whereas methods similar to those used to identify transcription terminators could be used to identify the 3′ end^51^. In addition, emerging methods involving the direct sequencing of RNA fragments in a sample (e.g., Nanopore direct RNA sequencingg)^52,53^ are expected to be compatible with PTV and should also allow the efficient detection of RNA phage termini and the characterization of their packaging mechanism.

We hypothesized that PTV should be able detect DNA ends not only in phages and viruses, but also in other biological entities, such as PICIs^54,55^ and other transducing agents with conserved DNA ends. For example, a recent study has described the packaging of non-random fragments of bacterial genomes inside phage capsids in more detail^40^. The authors of this study showed that the precise packaging of bacterial DNA is made possible by the presence of sites resembling phage pac sites at multiple locations throughout the host genome. Until recently, gene transfer agents (GTA), which are phage-like particles, were thought to package bacterial DNA in a random fashion only, but recent studies have shown that certain GTAs (i.e., those in the dinoflagellate-associated bacterium *Dinoroseobacter shibae*) can package non-random DNA fragments, presumably also using a headful packaging mechanism^56^. Like phages, these entities are also of great interest, as they are increasingly alleged to be involved in horizontal gene transfer, host adaptation, and bacterial virulence^57,58^. The ability to define the exact start and end of these sequences by identifying the termini should help in more precisely defining and characterizing these particles and their role in various ecosystems.

Going forward, the detection and thorough characterization of viral contigs in metagenomic datasets will benefit from the use of a combination of tools based on various approaches^59,60^. In summary, PTV is a new and distinct tool for the high-throughput characterization of phage genomes that should substantially accelerate the comprehensive study of the virosphere.

## Materials and methods

### Origin of samples, library preparation, sequencing, and contig assembly

Three sequence datasets were used in this study: (i) a simulated mock virome containing reads collected from five reference phages sequenced in our previous study (Lambda, HK97, T7, P1, Mu)^27^, in which the mock virome was generated by randomly subsampling 2 × 10^6^ paired-end reads from each phage dataset and grouping them in a single forward and reverse read file (1 × 10^7^ paired-end reads in total), (ii) a human gut virome sequencing dataset (SRR4295172) from a previously published study^37^, and (iii) a human gut virome sequencing dataset generated for this study. For this study, viral particles were separated and purified as follows: 500 mg of fecal matter was resuspended in sodium citrate solution (50 mL) and centrifuged at 300×*g* for 10 min at 4 °C. The supernatant was recovered and centrifuged at 5000×*g* for 45 min at 4 °C to pellet the bacteria. The supernatant was again recovered and diluted (1:5) with cold SM buffer (200 mM NaCl, 10 mM MgSO_4_, 50 mM Tris pH 7.5) and filtrated using 0.45 μm and 0.2 μm polyethersulfone membranes. For viral particle precipitation, PEG 6000 was added to a final concentration of 10% and the samples maintained at 4 °C overnight. The following morning, samples were centrifuged at 6000×*g* for 1 h at 4 °C and the pellets resuspended in 2 ml cold SM buffer and treated with an equal volume of chloroform. After centrifugation at 15,000×*g* for 5 min at 4 °C, the aqueous phase containing viral particles was recovered. SDS (0.5% final concentration) and proteinase K (20 mg/mL) were added and the samples incubated for 3 h at 65 °C. DNA was extracted using phenol chloroform, precipitated with ethanol, and resuspended in 10 mM Tris–HCl. DNA was sonicated using a Covaris S220 apparatus. The sequencing library was prepared using a TruSeq Illumina kit, 2 × 75 bp paired end, and sequenced on a NextSeq550 Illumina sequencer. The run generated 102,581,131 paired-end reads (15.6 Gbases total). The sequence dataset was deposited under SRR11177648.

### Read quality control and virome assembly

To generate a proper assembly of all virome datasets, raw reads were trimmed and cleaned using AlienTrimmer v.0.4.0^61^ and the NextFlex PCR Free adapter list, with the following options: -l 30. Cleaned reads were then assembled using metaSPAdes v.3.10.0^62^ with the following parameters: --meta -k 21,33,55,77,99,127 --threads 12. Contigs ≥ 10 kbp were retained for further analyses.

### Prediction and detection of phage contigs

Assembled contigs were analyzed for viral/phage detection using three different popular user-friendly software programs: MetaPhinder v.2.1^30^, VirSorter v.1.0.3^29^, and PHASTER (using multiple separate contigs option)^31^. All software was used with the default parameters.

### PhageTermVirome analyses

PTV v.4.0.0 was run in the paired-end mode with the default options for all datasets analyzed in this study.

### Phage contig annotation

Selected contigs were annotated with PROKKA v.1.14.0^63^, run locally, using the PHASTER curated database as the primary source of trusted phage proteins (as of December 22, 2020), with an E-value threshold of 10^−3^.

### Protein network analysis with vConTACT2

Network analysis with vConTACT2 v.0.9.22 was performed using three reference contig databases (GVD^8^, ViralRefSeq V.97^41^, and a database of curated virus-like particles from the mouse from Duerkop et al.^42^. The visualization of the protein-sharing network was done using Cytoscape software v.3.1.1; http://cytoscape.org/)^64^. The edge-weighted spring-embedded model was used to position genomes sharing most protein clusters in proximity to one another.

### Nucleotide sequence accession numbers

The raw read data of the human fecal virome from this study was deposited in the sequence read archive (SRA) under the accession number SRR11177648.

## Data Availability

PhageTermVirome source code is available at GitHub as a standalone software written in python3 (https://gitlab.pasteur.fr/vlegrand/ptv). A conda virtual environment is distributed with the software and all installation instructions are specified in the included “README.txt” file. Other datasets generated during the current study are available from the corresponding author on reasonable request.
