# Author Correction: High-throughput identification of viral termini and packaging mechanisms in virome datasets using PhageTermVirome

**DOI:** 10.1038/s41598-022-12064-0

**Published:** 2022-05-09

**Authors:** Julian R. Garneau, Véronique Legrand, Martial Marbouty, Maximilian O. Press, Dean R. Vik, Louis-Charles Fortier, Matthew B. Sullivan, David Bikard, Marc Monot

**Affiliations:** 1 Biomics Platform, C2RT, Institut Pasteur, 75015 Paris, France; 2 Infrastructure et Ingénierie Scientifique, Institut Pasteur, 75015 Paris, France; 3 Institut Pasteur, Unité Régulation Spatiale des Génomes, UMR 3525, CNRS, 75015 Paris, France; 4 Phase Genomics Inc, Seattle, WA 98109 USA; 5 Department of Microbiology, Ohio State University, Columbus, OH 43210 USA; 6 Faculty of Medicine and Health Sciences, Department of Microbiology and Infectious Diseases, Université de Sherbrooke, Sherbrooke, QC J1E 4K8 Canada; 7 Département de Microbiologie, Institut Pasteur, Groupe Biologie de Synthèse, 75015 Paris, France

Correction to: *Scientific Reports* https://doi.org/10.1038/s41598-021-97867-3, published online 15 September 2021

The Funding section in the original version of this Article was incomplete.

“This work was supported by the French Government’s program Investissement d’Avenir program; Laboratoire d’Excellence ‘Integrative Biology of Emerging Infectious Diseases’ [ANR-10-LABX-62-IBEID] and the Natural Sciences and Engineering Research Council of Canada (NSERC #341450-2010). Biomics Platform, C2RT, Institut Pasteur, Paris, France, is supported by France Génomique (ANR-10-INBS-09-09) and the Infrastructures de recherche en biologie, santé et agronomie (IBISA).”

now reads:

“This work was supported by the French Government’s program Investissement d’Avenir program; Laboratoire d’Excellence ‘Integrative Biology of Emerging Infectious Diseases’ [ANR-10-LABX-62-IBEID] and the Natural Sciences and Engineering Research Council of Canada (NSERC #341450-2010). Biomics Platform, C2RT, Institut Pasteur, Paris, France, is supported by France Génomique (ANR-10-INBS-09-09) and the Infrastructures de recherche en biologie, santé et agronomie (IBISA). M.M. and J.G. were supported by the “CDPhages” ANR JCJC Grant from ANR-18-CE35-0011.”

The original Article has been corrected.

